# Dental Education—Rejoinder

**Published:** 1884-08

**Authors:** Ferdinand J. S. Gorgas

**Affiliations:** Dean of Dental Department University of Maryland


### DENTAL EDUCATION.

The July number of the INDEPENDENT PRACTITIONER contained an article entitled "Dental Education," which purported to be a

"reply" from the faculty of the Baltimore College of Dental Surgery to an article, on the same subject, from Dr. B. Merrill Hopkinson, which appeared in the June number of the PRACTITIONER, charging the faculty of the Baltimore College with granting diplomas in a very irregular manner, to persons who had never attended the lectures, demonstrations, etc., of their school. This "reply" to Dr. Hopkinson's charges, all of which we believe to be strictly correct, far from exonerating the faculty of the Baltimore College, consisted wholly in an unprovoked attack upon the Dental Department of the University of Maryland, which had nothing whatever to do with Dr. Hopkinson's article.

In the first place, Dr. Hopkinson, before he wrote the article in question, and subsequent to the publication of the catalogue, had resigned his position of Demonstrator in the Dental Department of the University of Maryland, and is not now connected with that institution. As a graduate of the Baltimore College of Dental Surgery, and being well acquainted with the manner in which his *alma mater* has been conducted of late years, Dr. Hopkinson deemed it to be his duty to place before the dental profession what he considered to be an abuse of power and an injury to the alumni of the old school; and he alone is responsible for the article containing the charges of irregularity, in the enumeration of which the half at least has remained untold. Under the impression, apparently, that abuse of another institution would shield them from condemnation, and also on account of their inability to furnish any excuse for what Dr. Hopkinson charges them with, the faculty of the Baltimore College have attempted to show that the Dean of the Dental Department of the University of Maryland was willing to do as their Dean did in regard to one West Virginia and the three New Jersey gentlemen, who received the Baltimore College diploma last March without attendance upon the lectures, demonstrations, etc. It is scarcely necessary to state that if it had been as easy to obtain the dental diploma of the University of Maryland as it was to obtain that of the Baltimore College of Dental Surgery, the gentlemen in question would to-day be ranked among the alumni of the former institution, as every one of them openly expressed his preference for the diploma of the University over that of the Baltimore College. So many inaccuracies occur in the "reply" of the faculty of the Baltimore College to Dr. Hopkinson's charges, and so abusive is it of the University, that I deem a simple statement of the facts in the case to be necessary. Dr. James G. Palmer, of New Brunswick, N. J., late President of the New Jersey State Dental Society, before the opening of the session of 1883–84 of the Dental Department of the University of Maryland, applied to me as its Dean to know upon what terms three gentlemen of his State Society, who had been practicing dentistry for eighteen or twenty years, and whose reputation was well established, could obtain the dental diploma of our University. Two of these gentlemen were C. A. Meeker, of Newark, N. J., and F. C. Barlow, of Jersey City, N. J. Some correspondence ensued between Dr. Palmer and myself, but no definite arrangement had been made, when the New Jersey gentlemen presented themselves early in the session and attended some of the lectures and demonstrations, having matriculated and received their tickets. At this interview the question arose as to the time these gentlemen could pass at the University, and it was finally arranged that they should in the course of two sessions meet the requirements of the University for graduation; their length of practice entitling them to present themselves for graduation at the end of one session. Such an arrangement was made on account of their many years of practice, their ability as dental practitioners, and their home business. I do not remember ever to have met the New Jersey gentlemen on a Sunday in accordance with an agreement to do so made on a Saturday, as is alleged by the faculty of the Baltimore College in their "reply." On the contrary, as I recollect the matter, the New Jersey gentlemen arrived in Baltimore on one occasion, on Sunday, and the same afternoon, without my being aware of their presence in our city, called at my office. I am not in the habit of doing business on Sunday, for, to the best of my recollection, in the course of an active practice now extending over twenty-five years, I have never even filled a tooth upon a Sunday. During an interview with the New Jersey gentlemen, at which it is alleged by the faculty of the Baltimore College that I alluded to Dr. Tiffany's determination to vote against them, it is only necessary for me to state that, although Prof. Tiffany is the Clinical Professor of Oral Surgery in the University of Maryland, he has no vote on the graduation of dental students; hence no such statement could have been made by me.

As regards the case of C. S. W. Baldwin, of Matawan, N.J., who also received a diploma last March from the Baltimore College on the same terms as did Messrs. Meeker and Barlow, when Mr. Baldwin applied to me by letter, the same arrangement was proposed to him as to the others, under the impression, conveyed by his letter to me, that he had been practicing dentistry for many years, and enjoyed the same reputation and standing at home and in his State Society as Meeker and Barlow. Afterwards, and prior to Mr. Baldwin's visit to Baltimore, on the occasion of an interview with Messrs. Meeker and Barlow, these gentlemen informed me that they objected to graduate with C. S. W. Baldwin, so that I determined to hold his case in abeyance until further investigation could be made. But as Mr. Baldwin never had a personal interview with me, no further action on my part was taken. The only reason why these New Jersey gentlemen applied to the Dean of the Baltimore College of Dental Surgery for diplomas, was that by doing so they could save the time they would be required to pass at the University, and graduate on easier terms. So far from promising light examinations and guaranteeing diplomas to two of these gentlemen, as the "reply" charges, I positively deny such a statement, for I plainly informed them, when they repeatedly urged me to arrange for easy examinations, that they must meet all the requirements exacted from the other students. During my connection with the Baltimore College, extending over a period of twenty-five years, and with the University of Maryland, I never, in any case, guaranteed a diploma, and never shall do so. I have also opposed the granting of diplomas to those who did not meet the requirements of the school, and when diplomas were awarded in the old Baltimore College to others than the members of the class in attendance at the time, it was to those who had many years before attended one session and had been in active practice ever afterwards.

But few exceptions to the above can be referred to, although such cases were not confined to the past fifteen years, as is alleged in the "reply," but extended over a period commencing with the organization of the school. Until the present Dean entered the faculty of the Baltimore College, only men of acknowledged ability were so favored, and a majority of the faculty decided the matter.

At the Sunday interview with Messrs. Meeker and Barlow, and in the presence of Dr. James G. Palmer, of New Brunswick, N. J., who will confirm this statement, the two former gentlemen gave me to understand that, through Dr. Fred. A. Levey, of Orange, N. J., a graduate of the late Maryland College, and an intimate friend of the present Dean of the Baltimore College, they, Meeker and Barlow, had been offered graduation in the Baltimore College of Dental Surgery on the easiest terms imaginable—what I, at the time, understood to be *no attendance whatever, and the payment of a mere fee:* in other words, anything, if these gentlemen would leave the University of Maryland, and go to the Baltimore College. I freely admit that, in consideration of the represented ability of Messrs. Meeker and Barlow, and at the earnest request of Dr. Jas. G. Palmer, whose kindness towards them was so poorly appreciated, I did offer these gentlemen some reduction in the time of attendance, but my letter to C. S. W. Baldwin, as published in the "reply," shows that these New Jersey gentlemen were required to attend portions of two sessions, making about three months instead of the four months second-course students generally attend the majority of the dental schools—a very different arrangement from that they made with the Baltimore College, *where their entire attendance did not extend over one or two days,* and that, too, at a time when the lectures and demonstrations of the session had closed. Supposing that I did promise easy examinations (which I have no right or power to do), as is alleged in the "reply," and which I have already positively denied, I leave it to the readers of this article to determine how honorable would be the conduct of men who, after earnestly soliciting such a favor, would use it to injure the one granting it.

As regards the case of T. H. Schaeffer, another graduate of the Baltimore College who received a diploma last March on the same terms as the New Jersey gentlemen, a brief statement will suffice: During the latter part of February, 1884, Mr. Schaeffer called at my office, and at once informed me that he had been sent to me by Dr. S. H. Williams, of Charlestown, W. Va., whose practice he was about purchasing, and that he wished to obtain a diploma from the Dental Department of the University of Maryland, which, according to what he said Dr. Williams had told him was worth a great deal more than that of the Baltimore College. I informed Mr. Schaeffer that he could not obtain a diploma during the present session, as it would be necessary for him to attend at least one entire session. Mr. Schaeffer had given me to understand that he had formerly been in practice for some ten years, but for a number of years, eight I think, had been engaged in the manufacture of agricultural implements, and that he must obtain a diploma in order to comply with the dental law of West Virginia. Mr. Schaeffer then asked me if I thought he could obtain a diploma at once from the Baltimore College. I replied that he must ascertain that from the Dean of that school.

Mr. Schaeffer did not, as alleged by the faculty of the Baltimore College in their "reply," *by mistake call upon Dr. Gorgas*, for he stated, during this interview, that some one had told him since he arrived in the city, that I was not connected with any dental college, and that he had had some trouble to find out who was the Dean of the Dental Department of the University of Maryland. Shortly after this interview with me, Mr. Schaeffer stated in one of the dental depots of Baltimore, that Dr. Gorgas had refused him a diploma, but he was going to obtain one from the Baltimore College, for the Dean of that school had told him he could return home and come again to the college in two weeks' time, in order to be examined. We leave the profession to judge of the nature of an examination such a man as this could pass, who had not been in practice for, at least, eight years, and had never devoted any time to the study of dentistry.

As regards the cases of J. F. Dowsley and F. A. Twitchell, these gentlemen called upon me at the University and stated that they had been attending the Baltimore College for some days, but, as I understood, had not yet matriculated. They also stated that they were not satisfied with the Baltimore College, and thought that they would be better pleased with the University, as they had attended some of its lectures, etc. I informed them that they were at liberty to attend, for a short time, our lectures and demonstrations, so that they could judge for themselves, as they had each passed one course at the Boston Dental College. One of these gentlemen, if not both, had a beneficiary scholarship to the Baltimore College. Several days after this they called at my office and matriculated, paying for their matriculation tickets (which they still hold), and when about leaving, desired to know if their examinations upon certain studies, passed at the Boston Dental College as juniors, would be received in the University. I informed them that we could not do so, and several days afterwards, during which time I did not again see them, they informed me by letter that they had made a more satisfactory arrangement with the Dean of the Baltimore College.

The above is a true statement of the cases referred to by Dr. Hopkinson in his article, and from the fact that the course pursued by the present government of what was formerly the *old* Baltimore College, has become public, the only defense offered by that faculty is a "vicious attack," altogether unprovoked, upon a rival institution. Unfortunately for the Baltimore College, since it passed under its present government, the same course (and one openly advocated by its present dean) has been pursued as in the late Maryland College. I have learned from good authority that some of the last graduating class of the Baltimore College, who had complied with its requirements as they appeared in the catalogue, were so greatly dissatisfied, that they complained of the faculty having graduated a number of men without attendance.

Finally: it is perfectly absurd to allege that any other motive except that of easy graduation, both as regards time and examinations, influenced all of the gentlemen whose cases are referred to by Dr. Hopkinson, in the choice of their *alma mater*, and the author of the "reply" must have had a vivid imagination when he inferred as the facts of these cases could only be known to the Dean of the Dental Department of the University of Maryland, hence, the latter had some part in bringing the charges. For his enlightenment it is only necessary to state that the facts of these cases were known throughout the entire country long before Dr. Hopkinson's article appeared.

The course pursued by the Dental Department of the University of Maryland, from its organization to the present time, has been open and above reproach; and every student who has obtained its diploma has strictly conformed to all its requirements as published in its annual catalogues, and any statement to the contrary can be easily disproved. Since the action of the State Boards at Niagara Falls, the University of Maryland has conformed to all the suggestions of that Board, and will endeavor to do so in the future.

FERDINAND J. S. GORGAS, M. D., D. D. S.,
Dean of Dental Department University of Maryland.

Personally appeared before me on this, the seventh day of July, 1884, Ferdinand J. S. Gorgas, M. D., D. D. S., and made oath that he truly and sincerely believes the above statement, to which his name is attached, to be strictly true.

Sworn to before me, this 7th day of July, 1884.

JOHN L. BAKER, J. P.

